# Transition to a new nursing information system embedded with clinical decision support: a mixed-method study using the HOT-fit framework

**DOI:** 10.1186/s12911-022-02041-y

**Published:** 2022-11-28

**Authors:** Yue Zhai, Zhenghong Yu, Qi Zhang, Wei Qin, Chun Yang, Yuxia Zhang

**Affiliations:** 1 School of Nursing, Fudan University, Shanghai, China; 2 Department of Nursing, Zhongshan Hospital, Fudan University, Shanghai, China; 3 Department of Information, Zhongshan Hospital, Fudan University, Shanghai, China

**Keywords:** Hospital information system, Clinical decision support systems, Usability, User-centered design, Nursing informatics, Qualitative research

## Abstract

**Background:**

Nursing information systems embedded with standardized nursing language and clinical decision support have been increasingly introduced in health care settings. User experience is key to the adoption of health information technologies. Despite extensive research into the user experience with nursing information systems, few studies have focused on the interaction between user, technology and organizational attributes during its implementation. Guided by the human, organization and technology-fit framework, this study aimed to investigate nurses’ perceptions and experiences with transition to a new nursing information system (Care Direct) 2 years after its first introduction.

**Methods:**

This is a mixed-method study using an embedded design. An online survey was launched to collect nurses’ self-reported use of the new system, perceived system effectiveness and experience of participation in system optimization. Twenty-two semi structured interviews were conducted with twenty nurses with clinical or administrative roles. The quantitative and qualitative data were merged using the Pillar Integration Process.

**Results:**

The average score of system use behavior was 3.76 ± 0.79. Regarding perceived system effectiveness, the score of each dimension ranged 3.07–3.34 out of 5. Despite large variations in approaches to participating in system optimization, nurses had generally positive experiences with management and technical support. Eight main categories emerged from the integrated findings, which were further condensed into three themes: perceptions on system content, structure, and functionality; perceptions on interdisciplinary and cross-level cooperation; and embracing and accepting the change.

**Conclusions:**

Effective collaboration between clinicians, administrators and technical staff is required during system promotion to enhance system usability and user experience. Clear communication of organizational missions to staff and support from top management is needed to smooth the system implementation process and achieve broader system adoption.

**Supplementary Information:**

The online version contains supplementary material available at 10.1186/s12911-022-02041-y.

## Background

The electronic health record (EHR) has become the mainstream of nursing documentation with the introduction of nursing information systems (NIS). Standardized nursing languages (SNLs) are a commonly understood set of terms used to describe the clinical judgments involved in nursing care [1]. Embedding SNLs into the NIS is essential for extraction, exchange and integration of nursing data across disciplines and institutions, achieving secondary utilization of nursing information [2]. Based on standardized data on patient history and nursing assessment results, clinical decision support (CDS) is made available to accomplish meaningful use of EHR and to assist health care professionals with decision-making [3]. SNL and CDS are among the top research priorities in nursing informatics [4]. Despite its potential to support and transform nursing practice by simplifying record-keeping, promoting standards-based practice, and giving timely access to information to aid decision-making [5], the introduction of NIS, does not necessarily lead to user adoption [6]. Research showed that NIS has changed the way nursing is practiced, with mixed findings identified in terms of information quality and access, documentation burden, time spent on patient care, communication and care coordination, quality of care and ultimately, nurse and patient satisfaction [7, 8], which have implications for administration decisions on the implementation of NIS.

Multiple technology acceptance theories recognize user experience as key to the adoption of health information technologies (HITs) [9]. HITs poorly adapted to the work context can cause contradictions to nursing workflows, compromising EHR usability [10]. Perceived poor EHR usability is associated with a higher level of emotional exhaustion among nurses [11], hindering system adoption [12]. Therefore, a user-centered design with the participation of frontline staff is needed from the pre-introduction to the postimplementation stage to improve system usability to facilitate user adaptation [13]. Based on the Information System Success Model and the IT-Organization Fit Model, the human, organization and technology-fit (HOT-fit) framework proposed by Yusof et al. [14] can be used to evaluate the influence of user attitude and skills, communication, leadership and an IT-favorable environment on HIT adoption. This framework has implications for anticipating and preventing implementation barriers from occurring and exploiting facilitators to successful HIT implementation [15].

Despite the increasing popularity of CDS, its provider uptake remains unsatisfactory, with a recent meta-analysis revealing the overall uptake of clinical decision support systems among 3607 providers to be as low as 34.2% [16]. Transition to a new NIS can be challenging and has been reported to cause changes in nurses’ routine practices, leading to emotional insecurity and stress [17]. A recent Dutch study showed that almost half of respondents experienced results worse than their expectations 7 months after the implementation of a structured and standardized EHR [18]. Research into the interaction between technology-related, dispositional, and contextual attributes during CDS implementation is worth exploring. However, to the best of our knowledge, no study has focused on these multi-layer interactions in the nursing context. This study reports on the implementation of a CDS-embedded commercial NIS (care direct) in a large tertiary hospital in China. Guided by HOT-fit framework, this study sought to address the following research questions: (1) What are nurses’ perceptions and attitudes toward Care Direct? (2) How does the implementation of Care Direct affect nurses' daily practice? (3) How do technology, organizational and human attributes affect user adoption of Care Direct?

## Methods

### Design

This is a mixed-method study using an embedded design in which qualitative data and quantitative data were collected concurrently with the former given priority. The mixed-method design was used because it enabled researchers to gain a deeper understanding of the topic of interest where different sets of data triangulated with each other, contributing to the credibility of the study findings [19]. In our study, the quantitative and qualitative data (survey findings, obervation notes and interview transcriptions) were integrated at the analysis level. This study was reported in accordance with the Good Reporting of a Mixed Methods Study (GRAMMS) checklist [20] (see Additional file 1).

### Care direct

Developed in accordance with the internationally consented standard for nursing clinical decision support systems in EHRs [21], Care Direct integrated the SNL, evidence-based nursing knowledge base and big data analysis resources, with all data contents completely disassembled into the minimun data set and encoded with the SNL, meeting the national requirements of healthcare data standardization in China. An illustration of the modules in Care Direct and algorithms for documenting patient care is provided in the Additional file 2: Fig. S1.

### Study context

**Fig. 1:**
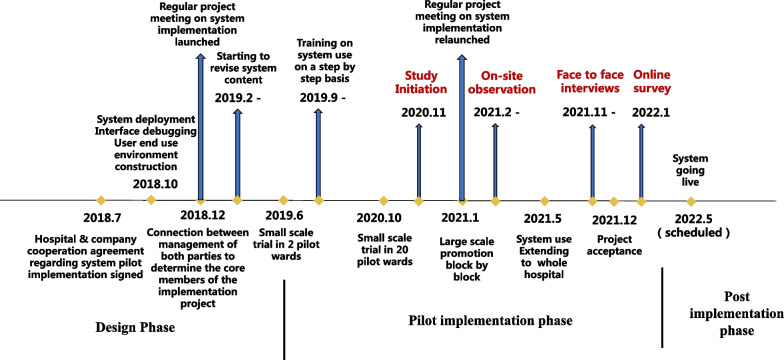
The timeline of Care Direct introduction

This study was conducted in a 2000-bed tertiary general hospital in Shanghai, China. Care Direct (the new NIS) has been running in parralel with the old hospital information system since its introduction in late 2018. At the commencement of this study in November 2020, Care Direct had been in pilot use (running in parallel with the old system) in 24 general medical-surgical wards for 1–17 months. The interoperability between Care Direct and exisiting hospital information systems was constantly optimizing during this study. The timeline of Care Direct implementation is shown in Fig. 1.

A project team was launched during the system design phase. A nurse manager (30 years in nursing) with previous experience of NIS implementation was appointed as the project champion. An on-site customer service personnel (2 years experience in HIT development) appointed by the vendor stationed in the hospital during workdays to respond to technical issues raised by nurses together with background technical staff as well as to communicate with nurse leaders regarding issues of system improvement. There was also a project advisor (30 years of experience in HIT development) and a technical director (11 years of experience in HIT development) who remotely connected with the team members while engaging in system development. No major changes in team members took place during the 3-year system implementation period except for the on-site customer service personnel.

### Sample

For the quantitative part, a cluster sampling was used to recruit nurses from 24 pilot wards to collect their perceptions and experience with Care Direct. For semi structured interviews, purposive sampling [22] was used to recruit nurses to ensure the diversity of experience, position, professional title, educational background and level of participation during system development in our study samples. The sample size was determined based on the principle of data saturation (no new category or concept appeared). Nurses on long-term leaves of absence, employed for less than 1 year and student nurses were excluded from this study due to limited access to or recent use of the NISs.

### Measure

The following instruments were used to collect nurses’ perceptions and experiences with Care Direct from various aspects: (1) human: the Nursing Information System Use Behavior Scale for nurses revised by Wen [23], (2) technological: the Clinical Nursing Information System Effectiveness Scale developed by Zhao [24] based on the Information System Success Model, and (3) organizational: a questionnaire about participation in system optimization developed based on the literature [25, 26]. For the third instrument, its construct and content validity were tested. The exploratory factor analysis extracted two principal components with eigenvalues greater than one, suggesting that the instrument can be divided into two factors: (a) nurses’ degree of participation in system development (5 items) and (b) nurses’ experience with participation in system development (8 items), explaining 75.47% of the cumulative variance. Seven experts specialized in nursing informatics were invited to evaluate the relevance of the items on a 4-point Likert scale, which resulted in a content validity index of 0.939. Revisions were made to three items according to expert opinions. The psychometric properties of the instruments in our study are shown in Table 1.

**Table 1:** Validity and reliability of the instruments used in this study

Tool	Type	No. of item	No. of dimension	Overall Cronbach’s α	Cronbach’s α for each dimension	CVI^a^
Nursing information system use behavior scale	5-point Likert	7	2	0.918	0.904, 0.922	0.978
Clinical Nursing Information System Effectiveness Evaluation Scale	5-point Likert	23	5	0.768	0.753–0.860	0.975
Questionnaire on the degree and experience of participating in system development	5-point Likert	13	2	0.928	0.914, 0.958	0.939

^a^Content validity index

### Data collection

#### Participant observation

From January to December 2021, the primary investigator (a research nurse with no clinical responsibilities) worked with the nurses and observed their use of Care Direct. The protocol of observation was developed based on the HOT-fit framework. On the wards, the observation focused on nurses’ interaction with Care Direct on which they performed routine tasks, especially its CDS options; meanwhile, nurses’ feedbacks regarding any technical issues (technological aspect) and capability and willingness to use Care Direct (human aspect) were also collected. We also paid attention to the leader role (of the charge nurse) in encouraging and standardization of the use of Care Direct within the unit (organizational aspect). (2) The primary investigator also joined a group chat involving core team members of system implementation and attended regular biweekly group meetings addressing system-related issues and work plans on system implementation as an observer to gain insight into the cross-level and multi-disciplinary collaboration during system optimization (organizational aspect).

#### Semi structured interviews

From October 2021 to January 2022, personal in-depth interviews were organized by the primary investigator to collect nurses' views and experience of Care Direct to verify and supplement the findings in the survey. An interview outline was prepared according to the research questions and existing frameworks (refer to Additional file 3). The topic guide was flexibly used to adapt to the different clinical roles of and responses from the interviewees by the primary investigator. Another investigator kept a note of the tones, gestures and facial expressions of the interviewee (s). The interviews were audio-recorded and transcribed verbatim within 24 h. Data analysis and collection were carried out simultaneously, and the interview outline was iteratively modified based on the data analysis results, which is conducive to the in-depth analysis of the themes and authenticity of findings [27]. Data reached saturation at 22 person-times. Information about the participants is shown in Table 2.

**Table 2:** Characteristics of interviewees (n = 20)

Demographics	N	%
**Work experience (year)**		
< 5	6	30.0
5–10	5	25.0
10–20	6	30.0
≥ 20	3	15.0
**Education**		
Associate	3	15.0
Bachelor	12	60.0
Master	5	25.0
**Role**		
Bedside nurse	13	65.0
Nurse specialist	3	15.0
Charge nurse	2	10.0
Nurse manager	2	10.0
**Participation in system development**		
Major^a^	6	30.0
Minor	14	70.0
**Core implementation team member**		
Yes	5	25.0%
No	15	75.0%

^a^Referring to having submitted requests or material regarding system implementation in written form to nurse leaders or technical staff

#### Questionnaire survey

An online questionnaire containing 48 required items (four items on nurses’ demographics) was used to collect nurses’ perceptions and experiences with Care Direct via Questionnaire Star (an online survey software). There were also two optional open-ended questions that prompted nurses to express their perceived advantages/disadvantages of Care Direct and expectations for its improvement. We asked the nurse manager of each block to distribute the questionnaire link to the charge nurse of each pilot ward after a clear explanation of the objective and inclusion and exclusion criteria of this study. Nurses were required to carefully read the instructions before completing the questionnaire. Respondents could submit the questionnaire only after completing all the required items, so there were no missing items; however, returned questionnaires with a response time of less than 90 s were excluded to ensure the validity of the results. A total of 384 nurses participated in the online survey, among whom 324 (84.4%) were included in the analysis. The demographics of the survey participants are shown in Table 3.

**Table 3:** Characteristics of survey respondents (n = 324)

Demographics	N	%
Working experience		
< 5	102	31.5
5–10	82	25.3
10–20	101	31.2
≥ 20	39	12.0
Education		
Associate	77	23.8
Bachelor	239	73.8
Master	8	2.4
Role		
Bedside nurse	277	85.5
Nurse specialist	23	7.1
Charge nurse	24	7.4
Willingness to use health information technologies in practice		
Not at all	2	0.62%
Somewhat	7	2.16%
Neutral	83	25.62%
Much	167	51.54%
Very much	65	20.06%

### Data analysis

Quantitative Data were imported into *SPSS* 22.0 for statistical analysis. The measurement data were described by the mean (standard deviation) or median (quartile) depending on the normality of the distribution. The counting data were described by frequency (percentage). The rank sum test was used to compare the differences in multigroup hierarchical data. On the qualitative branch, Investigators read through the observation notes and interview transcriptions several times to familiarize themselves with the contents.

**Fig. 2:**
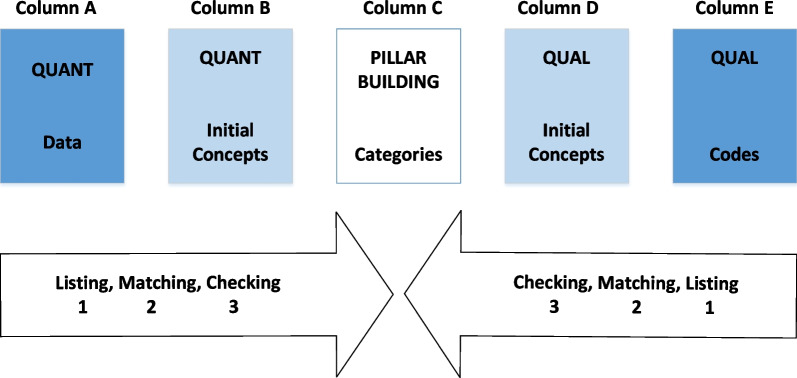
An illustration of the PIP [28]

The two sets of data were merged at the interpretation level using the Pillar Integration Process (PIP) proposed by Johnson et al. [28], presented in the form of a table including five row headings, quantitative data, quantitative concepts, categories, qualitative concepts and qualitative codes (Fig. 2). The PIP is a four-step process [28]: listing, matching, checking and pillar building. The categories emerged from the PIP were further integrated into themes using inductive reasoning and a final framework was formed.

### Trustworthiness and rigor

To enhance the trustworthiness of this study, member checking was employed to paraphrase and summarize the participants’ statement to ensure the intended meaning was accurately conveyed [29]. Interview transcripts were also returned to the interviewees within 24 h for verification, which ensured dependability. Transferability to other contexts was assured by describing the context, purposive selection of participants with different characteristics and using the PIP for conducting data analysis. Confirmability was guaranteed by various approaches to data collection and triangulation of multiple data sources guided by a biostatistician as an external auditor on study design, instrument development, data reconstruction and synthesis. To reduce the investigators’ own bias on the research process and results, constant self-reflexivity was employed during data collection and analysis to eliminate the possible interference of personal emotions and opinions with the research results [30]. 

## Results

### Quantitative results

#### System use behavior

The average score of system use behavior was 3.76 ± 0.79, with most respondents (53.40–73.46%) responding “agree” or “strongly agree” for each item. Refer to see Additional file 4: Table S1 for detailed results.

#### Perceived system effectiveness

The average scores of items in the five dimensions are as follows: system quality: 3.23 ± 1.00, information quality: 3.34 ± 0.98, service quality: 3.23 ± 17.69, user satisfaction: 3.07 ± 0.97, net benefit: 3.07 ± 1.05. Among the 23 items, the proportion of participants responding with “strongly agree” and “agree” ranged from 23.15 to 61.73%, with the median percentage being 36.73%. Refer to Additional file 4: Table S2 for the responses to each item.

#### Experience of participation in system development

Users' participation in NIS development was medium, with the percentage of participants responding with “very often” and “often” < 50% for all items (Table 4). There were significant differences in nurses' participation in different system improvement approaches (*χ*^2^ = 34.097, *p* < 0.001).

**Table 4:** Participating in system optimization: approaches and user experience (n = 324)

Items	Options/responses^c^			*χ*^2^
	1	2	3	
*Approaches to participating in system development*^a^				34.097^c^
I demonstrate system use and put forward relevant requests to technical staff on site	121 (37.35%)	89 (27.47%)	47 (14.51%)	
I participate in the system development group meetings led by management as a user representative	108 (33.33%)	76 (23.46%)	32 (9.88%)	
I put forward system-related problems and improvement requests on the online shared documents	106 (32.72%)	72 (22.22%)	42 (12.96%)	
I put forward system-related problems and improvement suggestions to technical staff through private WeChat / WeChat group	108 (33.33%)	70 (21.6%)	34 (10.49%)	
I put forward system-related problems and improvement suggestions to the designated nurse responsible for system development	101 (31.17%)	104 (32.1%)	50 (15.43%)	
*Experience of technical support during system implementation*^b^				32.781^c^
When I want to feed back the problems of the system, I know who to feed back to	32 (9.88%)	136 (41.98%)	153 (47.22%)	
The management take our opinions and experience seriously	37 (11.42%)	126 (38.89%)	156 (48.15%)	
Technical staff listens to our feedback	51 (15.74%)	127 (39.2%)	143 (44.14%)	
Technical staff are aware of our suggestions and experiences	58 (17.9%)	132 (40.74%)	126 (38.89%)	
Technical staff modify and improve the system according to our needs	50 (15.43%)	136 (41.98%)	129 (39.81%)	
Improvement needs can be implemented quickly enough	65 (20.06%)	135 (41.67%)	99 (30.56%)	
According to my experience, our feedback can be conveyed to technical staff all the way up	47 (14.51%)	144 (44.44%)	130 (40.12%)	
Technical staff go deep into the clinical setting to understand user requests	74 (22.84%)	133 (41.05%)	98 (30.25%)	

^a^1 = Sometimes, 2 = Often, 3 = Very often. The rest participated Very little/Not much

^b^1 = neutral, 2 = agree, 3 = strongly agree. The rest disagreed/strongly disagreed

^c^*p* < 0.001

Nurses had an overall positive experience with respect to communication with the management and technical staff during system development, with the percentage of participants responding with “strongly agree” and “agree” > 70% (Table 4). There were significant variances in nurses’ experience regarding different aspects of technical support (*χ*^2^ = 32.781, *p* < 0.001).

### Merging qualitative and quantitative findings

The qualitative data were merged with the quantitative data via the Pilar Integration Process (Table 5).

**Table 5:** Merging quantitative and qualitative data using the PIP

QUAN		Category	QUAL	
Data	Concepts		Concepts	Codes
Net benefits: only 26.59% of respondents agreed/strongly agreed that “The system reduced the time required to complete the tasks.” (*Below average score*) Net benefits: 31.5% of respondents disagreed/strongly disagreed, and 33.53% were unsure that “The system could better streamline the process of nursing practice.” (*Below average score*)	Increasing the complexity of nursing documentation	Record templates increasing documentation burden	Limited value of nursing process-based templates	(The admission assessment is) too complicated. These things may take a long time to complete for each patient, and they will feel “why the nurse needs to ask me these things, which has little to do with my disease”. (P9, nurse) Some patients are (in) particularly good (condition). They have no past history or anything, but by default, you have to include two (nursing diagnoses), so you just add them by yourself… In fact, it’s meaningless. It’s about completing the process and you can't leave one stage unfinished. (P13, nurse) (Shift handover summary) contains too much stuff. We all pay attention to the key points during handover, but we won’t be able to find the key points in a pile of texts. For example, I hope to see clearly that this patient has a catheter, but it’s is buried in a paragraph of texts which can't be found (easily). (P6, nurse)
System quality: Only 26.59% of respondents agreed/strongly agreed that “The system can flexibly switch between various screens.” (*Below average score*)	Inflexibility switching between various screens		Documentation burden caused by navigation between screens	It is cumbersome to document the I/O, the time needs to be re selected for each input (for each patient). (Survey response) Not all content can be integrated in one interface. (Survey response) Care Direct requires nurses to shift between various screens to complete observation and care items as opposed to documenting directly next to the narrative texts as in the paper records. (Observation notes)
Information quality: 14.81% of respondents disagreed/strongly disagreed, and 42.28% were unsure that “The information provided by the system is continuous and dynamic, which can reflect the change process of the patient's condition.” (*Below average score*)	Information not reflective of the dynamic change of patient condition	Problematic Information linkage	Unavailability of information linkage within the system	The system can capture the abnormal data, for example, the labs, but it will not give you suggestions… Generally, you will not reassess the patient unless there is a change in his care demand. But the patient's situation is actually a dynamic process. This thing (system) is dead, it is not that dynamic… In fact, I see the doctors’ (system) doing quite well, for example, when the labs report that the patient’s blood potassium is high, it will remind you with suggested actions, but ours won't and you have to add it manually. (P13, nurse)
Information quality: 19.13% of respondents disagreed/strongly disagreed, and 42.28% were unsure that “The system can obtain the required information within the timeframe required of nursing practice.” (*Below average score*)	Information not generated in a timely manner to support the user		The timing of interventions generated on the schedule not conforming to practice routines	Why would *preoperative education* be triggered on the night shift the day before operation? Generally, we do this education on the day shift before the operation, and the patients who do surgery on Monday are educated on Saturday. (Survey response) Now the doctor makes a new diagnosis, it (the system) will generate a care bundle corresponding to the diagnosis and inexplicably auto-select all the corresponding interventions… directly reflected in the schedule… It is completely inexplicably presented. Then you have to go back to the care planning module to choose what you need or delete them whole… (P9, nurse)
Information quality: 17.28% of respondents disagreed/strongly disagreed, and 35.80% were unsure that “The information provided by the system is consistent with the actual situation and there are no errors in the record.” (*Below average score*)	Information generated by the system lacking accuracy		The error-prone information generated by the system adding to documentation burden	We're worried that we cannot control the system-generated record sheet after it goes live… We can't see the final appearance of what we document in the system. If we want to see it, we have to generate it separately… You won’t be able to check on every shift to see whether the documentation for each patient is right. I don't have time to check. (P8, nurse specialist)
Net benefits: 40.43% of the subjects agreed/strongly agreed that “The system had the ability of analysis and prediction in the nursing process.” (*Above average score*)	Analysis and prediction function of the system	Value of CDS	Various perceived benefits from CDS	When I need to raise nursing problems for my patient, CCC (the system) automatically jumped out (recommended) her related nursing problems… I just need to choose among them without thinking about them on their own. (P10,nurse) For staff who have worked for many years, they have formed a working mode. If they know what to do at each point on the shift, they won’t follow this system and may have formed an inherent thinking. It's actually quite difficult to change this inherent thinking. If you ask us to completely follow the care plan, we may feel that we won’t be able to do it. (P8, nurse specialist)
			Serving as a reminder	Maybe it provides more options, which can give us a hint. Sometimes we can't think of it. There's a hint in the system. (P14, nurse) Sometimes it's not the nurses deliberately don't implement it (the tasks), but they really forget about it, right? They may pay more attention to dealing with physician orders or do treatments, but they will neglect some other interventions. Then we (the system) push them (all the schedules) out to remind them, which can guarantee the care quality. (P16, nurse manager, project champion) The schedule can better remind nurses of some hidden nursing interventions that are easy to be neglected. (Survey response)
Service quality: 18.52% of respondents disagreed/strongly disagreed, and 38.58% were unsure that “The training on system use could meet the needs of clinical practice.” (*Below average score*)	Training on system use not meeting user needs	Insufficient training on system use	Inefficiency of training organization	At that time, we went over there (the venue) for centralized training. The place was large, (we sat) too far away and could not see clearly, and it was noisy on the scene… (P3, nurse) He just told you in a very general way what there are in each module…I can see these as long as I click them by myself. Anyway, it was of little use. What I need is after the patient come back from surgery, what are the actions to be taken in the system step by step. (P3, nurse) Wasn’t it first carried out in the pilot places (wards)? Staff at other departments didn't know about it. I learned from the colleagues after I went to the pilot place (ward). (P5, nurse) It (the training) was good, but I don’t want (the leaders) to force nurses to take the training during rest time. It can be made into a paper version or an electronic version, distributed to the workgroup and learn by ourselves. (P10, nurse)
Service quality: 29.94% of respondents disagreed/strongly disagreed, and 37.96% were unsure that “Technical staff can understand the special needs of nurses.” (*Below average score*)	Technical staff having difficulty understanding user needs	Cooperation between nurses and technical staff	Interprofessional barriers	When communicating with technical staff, my strongest feeling is powerlessness. They can't understand many of the requirements we put forward, and the final product is not what we want. (P20, nurse manager)
Service quality: 11.73% of respondents disagreed/strongly disagreed, and 38.27% were unsure that “Technical staff sincerely and timely solve the problems encountered during system use.” (*Above average score*)	Technical issues not solved quickly enough		Nurses disenchanted with system improvement	When the problems you reported repeatedly not solved after a long time, it's a heavy blow to us… (P2, charge nurse) Just don’t bother to talk to them… Some problems may be raised at the beginning, but you found that after a long time, why it still hasn’t been solved? There's just no big progress. (P6, nurse)
Experience of technical support: 81.79% of respondents agreed/strongly agreed that “Technical staff modify and improve the system according to our needs”	Technical staff value user requests		Recognizing efforts made by technical staff	Certainly, there is no way to solve all the technical problems in the short term. We can see that the engineers have been making modifications and it (the system content) is closer to what we want, but it has not completely reached that point, there is still a little distance. (P17, nurse)
Experience of technical support: 86.69% of respondents agreed/strongly agreed that “The management take our opinions and experience seriously”	Management value feedbacks from the nurses	Leader role	Leader Supervision	We do what the leader says about. (P1, nurse) Especially when the leader (charge nurse) comes to ask you why this is not properly documented (in the system), we will give her an explanation. Then she will ask you to display it (in the system), try it, and then she will also operate it herself (to identify the problems with the system) so she could report it to the higher (management). (P5, nurse) Now the charge nurse will review this (the nursing record exported from the new system) and point out our mistakes. After all it is still in the pilot stage and has not been included in nursing quality assurance, but it will certainly be included later. (P13, nurse)
			Rational arguments	I have been telling them that this is about a state of mind. This (the system) is a new stuff. It would probably be better if you could do it with a learning and accepting mindset. (P16, nurse manager, project champion)
Experience of technical support: 84.61% of respondents agreed/ strongly agreed that “According to my experience, the problems I reported can reach technical staff all the way up”	A smooth feedback channel	Cross-level collaboration	Communication between nurses on different levels	There is no problem in our communication with ward nurses. I can clearly their requests. After the nurse gives me feedback, we will check whether it is minority opinions or majority opinions, and then we decide to communicate with the technical staff. (P20,nurse manager) Sort out (documentation requirement) according to the practice routines in our hospital. For example, the catheters, each head nurse sorted out (the types of) catheters in use in her unit. (P15, charge nurse) Problems were discussed in person among nurse leaders and the project champion and common problems summarized before being put forward on the project meetings. (Observation notes) During 13-month observation period, the system underwent 45 formal version upgrades, with 103 system-related bugs repaired, 131 system optimizations achieved and 50 new functions & contents added. (Observation notes based on documents review) In terms of coordination and communication, we all go in the same direction. However, I think there must be a consistent way (of communication). (P16, nurse manager, project champion) Top management did not attend discussion sessions regarding system content revision as proposed by her despite repeated invitations made by the project champion; therefore, she was unconscious of the appropriateness of content revision. (Observation notes)
Information quality: 43.21% respondents agreed/strongly that “The information provided by the system can meet the types and contents of information required of nursing practice.” (*Above average score)*	Integrity of information content provided by the system		Positive outcomes of collaboration	The (system) function has also been constantly improving, which is indeed much more convenient. (Survey response) It (the system) has an option for basically anything you want. This is the biggest improvement. At the beginning, it may not have so many options, that is, for example, the location of the catheter is not so clear. Now it is basically perfect, and this progress is a huge one. (P3, nurse)
NIS use behavior: 53.40% of the subjects agreed/strongly agreed that “I have get used to the new system”	Getting used to the system	Accepting the new thing	Different levels of adaption to the system	Our old system is very straightforward, while the new system requires us to demonstrate our clinical thinking processes, which requires a certain amount of time to adapt. (P20, nurse manager) For the less optimized software at the initial stage, it may affect everyone's perceptions of use, but now that we are gradually on the right track, I think we have adapted to its use. (P7, nurse specialist) I have accepted it very much now. (P1, nurse) Now there are too many systems, and the work is a little complicated and we feel a little confused. (P12, nurse) I don't know what will happen later on. If I don't need to write the paper ones in the future, just use the electronic system, I think it will probably be OK after extended use. (P18, nurse)
NIS use behavior: 70.37% of the subjects agreed/strongly agreed that “I actively learn the existing functions of the system”	Learning system use strategies		Proactively learning system use	If I don’t know how to handle it, I will definitely take the initiative to learn, because it relates to my clinical work. (P6, nurse)

### Integrated findings

Eight main categories emerged from the integrated findings, which were further condensed into three themes: (a) perceptions on system content, structure and functionality; (b) perceptions on interdisciplinary and cross-level cooperation; and the overarching theme (c) embracing and accepting the change.

### Theme 1: perceptions on system content, structure and functionality

#### Record template increasing documentation burden

**Fig. 3:**
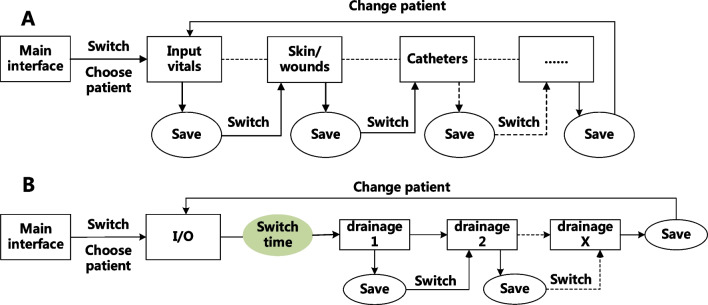
Illustration of the steps of record-keeping in care direct (**A** documentation of observation items for acute postoperative patients; **B** documentation of fluid output)

The documentation framework based on the nursing process in Care Direct is not conducive to reflecting the whole picture and the dynamic changes of patient conditions, which compromised the communication of information among nurses. With the large amount of information to be collected in the past history module and some items not being closely related to the patient's conditions, nurses felt it a mere formality to meet the documentation requirement, which may have caused negative experiences for the patient. Moreover, the lengthy overview containing a large amount of irrelevant information generated by Care Direct failed to meet nurses’ information needs; therefore, Care Direct was rarely referred to as a source of information exchange. The need to frequently switch between screens also added to nurses’ documentation burden and increased the risk of missing information (Fig. 3 and Table 5).

#### Suboptimal information linkage

Negative experiences regarding issues with information linkage in Care Direct were frequently referred to in our study. While Care Direct achieves automatic linkages across each stage of the nursing process through CDS with a preset criteria for reassessment according to patients’ care demands, it cannot give decision support in response to changes in patient condition such as abnormal vital signs, abnormal laboratory results, and documented signs/symptoms. Although the system allows users to add nursing problems and update care plans manually, nurses would not bother to do so based on the findings from on-site observations and record review by the investigators.

Many decision support rules of Care Direct were triggered by medical diagnoses or medical orders generated in the old system, but the push timing did not conform to the clinical workflow, which required nurses to manually add or delete schedules according to work routines and actual situations. Problems with information linkages also hindered the system from generating accurate nursing records based on user actions. While Care Direct allows users to view and edit the nursing documentation, heavy clinical tasks kept nurses from spending much time on verification (Table 5).

#### Value of CDS

Care Direct automatically links the five steps of the nursing process in the form of decision support, as opposed to the separate status of different components in the old system. The value of system decision support is also reflected in the daily reminder for nurses. Clinical nursing is composed of numerous tasks and the schedule generated by the system can remind nurses of tasks they tend to neglect, ensuring delivery of quality care. Nevertheless, nurses had different levels of perceived system benefits. Some nurses thought that CDS could supplement their clinical reasoning to facilitate decision-making; others, however, viewed CDS as a disruption to their inherent thinking and work habits and thus were reluctant to follow the system’s recommendations (Table 5).

### Theme 2: perceptions on interdisplinary and cross-level collaboration

#### Insufficient training on system use

Shortly after the initiation of pilot use, training on system use was organized to familiarize nurses with Care Direct. To ensure that every nurse on each pilot ward attended at least once, the training was carried out during the lunch break every weekday. The way the training was organized, however, was inconducive to the learning efficiency of participating nurses. Moreover, the content of training sessions provided limited support for nurses. Due to the lack of contexts, it failed to solve the problems encountered by nurses during daily use of the system. Lack of continuity of training was also a problem for nurses. As Care Direct was only piloted in general wards, some junior nurses who later rotated from other units missed the training sessions (Table 5).

#### Collaboration between nurses and technical staff

Due to interprofessional barriers between nurses and technical staff, cooperation between the two was suboptimal. Lacking understanding of the nursing workflow, technical staff had trouble understanding the requests made by nurses, leading to misalignment between user expectations and system outcomes. The survey showed that among all aspects of nurses' experience with participating in system development, their satisfaction with the speed of request solving was the lowest. Unmet needs led to nurses’ disenchantment, rendering them unwilling to provide additional feedback; instead, nurses chose to adapt to the imperfections of the system. Nevertheless, nurses did recognize efforts made by the technical staff and understood that the heavy workload they were facing hindered them from handling user requests in a timely manner (Table 5).

#### Leader role

Most nurses agreed that the management took their suggestions and experiences seriously. Support from management is key to user adoption. Although nursing documentation under Care Direct was temporarily out the scope of quality audit, to ensure nurses smoothly transition after it goes live, charge nurses on the wards communicated with staff nurses about the omissions identified from regular record review to determine whether these were caused by the user or technical defects, which urged nurses to establish positive system use behavior and was also conducive to system optimization (Table 5).

#### Cross-level collaboration

**Fig. 4:**
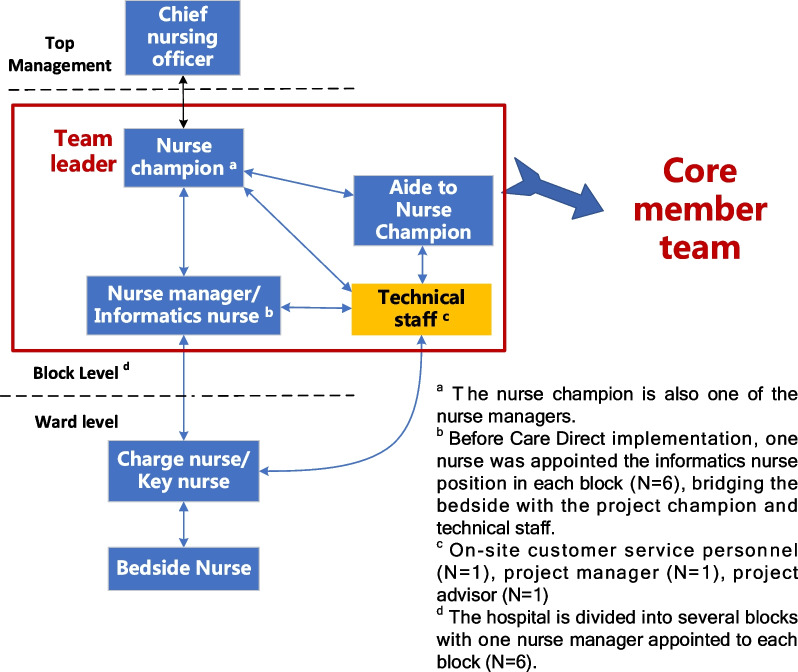
A bottom-top approach to raising requests and feedbacks

Care Direct was a commercial NIS purchased from a third party without adequate input from frontline nurses at the design phase; therefore, local adaptation was needed to improve its suitability. A feedback pathway involving the project leader with previous experience in NIS development (nurse champion), nurse managers/informatics nurses at each block, ward nurses and technical staff was established (Fig. 4); meanwhile, a project team including the core members involved in system implementation was launched. Taking a bottom-up approach, each ward reported system-related problems and improvement requests to management, which would be gathered by the nurse champion. Problems were discussed in person among nurse leaders and common problems were summarized before being put forward on the project meetings. Relevant materials were prepared by the nurse leaders and submitted to the technical staff to initiate improvements. Relying on the wisdom of nurses at all levels, the integrity of system content and function was constantly improving to streamline nursing workflow  (Refer to Additional file 5 for details of upgrades in each system module) (Table 5).

Despite the overall positive outcomes of cross-level collaboration, it was not without barriers. Designation of responsibilities seemed to be suboptimal among the project team, rendering the project champion to face significant pressure during system implementation. Sometimes, efforts and outputs made by the project champion awaiting feedback were not responded by other team members or top management, possibly due to heavy administrative workload, which also led to her frustration (Table 5).

### Overarching theme: embracing and accepting the change

Since the implementation of Care Direct was decided by the management, its use was mandatory, and competence in handling the system would be part of nursing practice. Therefore, some nurses explored system functions and gradually became proficient users. Most survey respondents agreed that they had become accustomed to the system. With routinized system use and improvement in system functionality, most nurses adapted to Care Direct and incorporated it into their daily practice. However, some participants who were lagging in HITs felt overwhelmed by the two NISs running in parallel and thus were slower to get on board with the new system (Table 5).

**Fig. 5:**
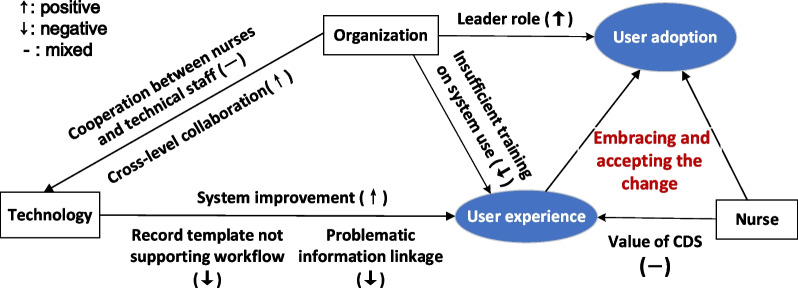
The proposed framework of user adoption

Overall, nurses generally took a rational position toward the benefits and hardships during their transition to a new NIS. While Care Direct was constantly adapting to the needs of users, users were also constantly adapting to Care Direct. A proposed framework demonstrating the relationships between the categories and themes is shown in Fig. 5.

## Discussion

The aim of this study was to investigate nurses' views and experiences during transition to a new NIS, focusing on the interaction of organization, technology and human attributes during system implementation. Findings from this mixed-method study revealed both positive and negative emotions related to system content, structure and functionality, interdisciplinary and cross-level collaboration. Despite nurses’ mixed emotions towards the implementation of Care Direct, they tended to integrate it into their routine workflow.

The results of this study showed that the lengthy documentation templates and suboptimal data linkage compromised the perceived usability of Care Direct. The introduction of SNL-based recording templates reduced the heterogeneity in free-text documentation, promoting data integration [2] and the quality of nursing documentation [31]. Notwithstanding their contribution to the integrity of nursing records, structured templates within the NIS failed to fully match the nurses' complex and dynamic workflow [32]. Fragmented information forced nurses to navigate between different interfaces to search for information, adding to their cognitive burden [33], as also reflected in our findings. Moreover, the restrictive nature of structured documentation framework has been criticized as compromising the accuracy of documentation [34, 35]; therefore, system-generated care plans and summaries were rarely referred to as a guidance to practice, but as a documentation requirement to serve administrative needs [36]. Considering the drawbacks regarding the clinical benefits of structured documentation framework, free-text input have been made available to supplement SNL-based documenation. Technical experts should be consulted to analyze the impact of Care Direct implementation on nurses’ information seeking and sharing practices and propose strategies to adapt it to the nursing workflow by, for instance, optimizing the relevance of the content within the summary/overview of care and shift reports generated by the system.

To ensure the clinical usefulness and user adoption of HITs, frontline nurses should take a dominant role during all phases of its implementation to voice their expectations [37]. In our study, the bottom-up feedback mechanism enabled all end users to express their concerns and expectations with Care Direct, contributing to broad participation. This hierarchical feedback system, however, deprived nurses of opportunities to sit at the same table with management and technical personnel, as demonstrated by the survey results. Our study also indicated that end users appreciated exchanging ideas with the nurses responsible for system development, which is consistent with previous findings [26]. Grounded in the clinical setting, informatics nurses acted as advocates for bedside nurses while working closely with technical staff to raise suggestions regarding usability issues with full consideration of the nursing workflow [38]. Before the introduction of Care Direct, the informatics nurse post was set up in our institution, with one nurse assigned to this position in each block, responsible for gathering and reporting system-related issues, assisting the project leader in drafting improvement strategies and connecting with technical staff.

The accessibility of technical support is an important factor affecting the implementation of HITs. In our study, contradictory findings were found regarding the perceived timeliness of technical support in the survey and interviews. In the survey, respondents provided an overall positive feedback based on the average score whereas the interviews revealed more negative experience. This was probably because some users tended to emphasize their negative experiences with system use due to its huge influence on their daily practice while selectively neglecting the positive ones. Another explanation is that they hoped their negative experience would raise concerns among investigators to guide future system improvement. A nationwide survey [26] conducted across Finnish public hospitals found that most clinicians perceived software vendors as being unresponsive to user feedback; however, technical staff had diametrically opposed views on these issues [25]. A possible explanation is that technical staff mainly interact with user representatives who are in administration positions and lack personal experience with end users’ pain points during daily use of the system, which was the case in our study, and there may be a gap between the user representative’s understanding and end users’ expectations. The lack of two-way communication between end users and technical staff is prone to negative emotions among users and the belief that their needs are not valued, leading to their disengagement with system implementation [39]. As the most direct method of information communication, in our study, on-site observation and demonstration of system use were frequently employed as approaches to identifying problems between end users and technical staff, which is also in line with previous studies [25, 26].

Support from leaders were well-received by nurses based on our investigation. During pilot use of Care Direct, the charge nurse in each unit generally took on the role of super user due to their high degree of participation in system development, and this produced moral effects to promote positive system use behavior in the whole unit. Nurse leaders play an important role in the promotion of HITs, as their support and supervision are imperative to leading nurses through resistance and doubt to achieve organizational change [40]. However, studies have reported that nurse leaders face significant obstacles in driving the implementation of HITs, such as insufficient understanding of the value of HITs due to limited informatics literacy, time constraints due to administrative tasks, and lack of support from top management, which hindered their ability to provide adequate support to nurses and make informed decisions about system improvement [41, 42]. In our study, despite the long-term commitment to HIT promotion and proactive leadership and partnership with technical staff demonstrated by the nurse champion during system implementation, barriers were encountered regarding training organization, interdisciplinary interaction and lack of engagement from top management. Therefore, implementation strategies are needed during future HIT implementation, and attention should be given to strengthen nurse leaders’ project management competencies [43].

User-perceived benefits are key to system adoption. As the end user of the new system, nurses' willingness to adopt Care Direct is largely related to their acceptance of it. Previous research [44] indicated that nurses will take the previous major change events as a reference to form their expectations for organizational change. We assumed that in the early stage of Care Direct implementation, the gap between system function and nurses’ expectations aggravated their negative response to the system, while the later acceptance came from their gradual adaptation to the system and the continuous improvement of system function. Despite their mixed feelings toward Care Direct, nurses tended to adapt to it rather than return to the original record-keeping modalities.

Finally, our study showed that nurses have mixed opinions about their perceived value of CDS. It is worth noting that the decision-making suggested by the CDS is to supplement rather than replace the professional thinking of nurses. Clinical experience is an important determinant of the perceived benefits from CDS. By using clinical intuition, senior nurses have a better grasp of the overall situation of patients and have internalized the nursing process into practice, thus having lower needs for practice guidance from the CDS [45]; fully following the clinical practice recommendations would reduce their autonomy [46]. Previous research [47, 48] showed that nurses rarely make decisions based on the CDS recommendation alone but combine subjective and objective patient information to identify the possible deviations of CDS and to reach more accurate and comprehensive decisions. Therefore, while enjoying the convenience brought by CDS, nurses still need to cultivate problem-solving skills by transforming readily available knowledge into improved care quality and patient outcomes [49].

### Implications for future research/practice

As with all HIT implementation, optimization of system structure and content should be an ongoing process with continuous input from both nursing and technical staff following its implementation. Technical issues regarding both software and hardware should also be solved by actively approaching hospital informatics personnel. From a safety perspective, the system should incorporate a double check mechanism to minimize the negative consequences of erroneous data linkage. To bridge the gap between nurses and technical staff, frontline nurses, who have deepest connection with the new HIT, should be given the opportunity to participate in learning sessions on nursing informatics to provide suggestions for interface customization. Technical staff should also be invited to immerse themselves in the clinical environment to familiarize themselves with the nursing workflow so that they will better understand user requests.

Nurses’ negative emotions are a common phenomenon during their interaction with the system. To mitigate the potential detrimental consequences of system-related negative emotions, during system pilot-run, management should pay attention to nurses’ additional workload related to system use and actively build connections with nurses to obtain their feedbacks. In future studies, structured observation checklists or nurse self-rated questionnaires could be used to investigate the impact of EHR on their care delivery to come up with strategies to avoid overburdening nurses.

### Strengths and limitations

This study has several strengths and limitations. First, our study recruited nurses from both clinical and administrative positions from purposive sampling where nurse leaders from the core member team shared their views on inter-disciplinary collaboration. However, insights from the project advisor and technical staff were lacking as their perspectives could complement with or possibly differ from those of nurses and could have been valuable to our study. Second, the cross-sectional nature of the quantitative branch precluded us from revealing the longitudinal changes of nurses’ perceptions and experience with system use over time; however, we were able to indirectly capture these changes from the qualitative responses. Third, this a mixed-method study guided by a theoretical framework, as reflected in the design of both quantitative and qualitative parts. Quantitative and qualitative data were innovatively triangulated with the Pilar Integration Process. However, we did not adequately excavate the possible contradiction between quantitative and qualitative results, which prevented us from drawing further inferences from the possible discordance.

## Conclusions

This study builds on the existing information technology acceptance models to show that the promotion of a NIS requires effective collaboration between end users, administrators and technical personnel to enhance system usability and user experience. Optimization of system structure and content and tackling technical issues should be an ongoing process with continuous input from both nursing and technical staff. Nurse leaders can exert a positive impact on nurses to facilitate system implementation by fostering relationships across disciplines. Frontline nurses should be given the opportunity to get involved in system development so that nursing practice will truly benefit from NIS. Future HIT use training should include clinical simulation sessions to better engage and truly benefit nurses. Aligning system implementation with broader organizational goals and support from top management is needed to smooth the transition process and achieve organizational level system adoption.

## Supplementary Information

**Additional file 1**. Good Reporting of A Mixed Methods Study (GRAMMS) checklist.

**Additional file 2: Fig. S1**. An illustration of the modules and operation procedures of Care Direct in client management.

**Additional file 3**. The topic guide (final version).

**Additional file 4**. **Table S1.** Responses to the NIS use behavior scale (n = 324). **Table S2.** Responses to the NIS effectiveness scale (n = 324).

**Additional file 5**. Table S3. Upgrade frequency of each module of the system from January 2021 to April 2022.

## Data Availability

The datasets used and/or analyzed during the current study are available from the corresponding author on reasonable request. The datasets supporting the conclusions of this article are included within the article and its additional files.

## Abbreviations

CDS: Clinical decision support
EHR: Electronic health record
HIT: Health information technology
HOT-fit: Human, organization and technology-fit
NIS: Nursing information system
PIP: Pillar integration process
SNL: Standardized nursing language
