# Supplementary material for: Transition to a new nursing information system embedded with clinical decision support: a mixed-method study using the HOT-fit framework
Source: BMC Med Inform Decis Mak. 2022 Nov 28;22:310. doi: 10.1186/s12911-022-02041-y (PMC9703774; doi:10.1186/s12911-022-02041-y)
Supplement: Supplementary file 1 — Additional file 1. Good Reporting of A Mixed Methods Study (GRAMMS) checklist. [file 12911_2022_2041_MOESM1_ESM.docx]

**Additional file 1. Good Reporting of A Mixed Methods Study (GRAMMS) checklist**

| **Guideline** | **Section: page** |
| --- | --- |
| Describe the justification for using a mixed methods approach to the research question | background- pg. 4 |
| Describe the design in terms of the purpose, priority and sequence of methods | Methods- design pg. 5 |
| Describe each method in terms of sampling, data collection and analysis | Data collection pg. 7-9  Analysis: 9-10, Table 5 |
| Describe where integration has occurred, how it has occurred and who has participated in it | Design: pg. 9-10 |
| Describe any limitation of one method associated with the present of the other method | Discussion pg. 20,23 |
| Describe any insights gained from mixing or integrating methods | Discussion: pg. 20 |

O'Cathain A, Murphy E, Nicholl J. The quality of mixed methods studies in health services research. J Health Serv Res Policy. 2008;13: 92-98.
