# Supplementary material for: Transition to a new nursing information system embedded with clinical decision support: a mixed-method study using the HOT-fit framework
Source: BMC Med Inform Decis Mak. 2022 Nov 28;22:310. doi: 10.1186/s12911-022-02041-y (PMC9703774; doi:10.1186/s12911-022-02041-y)
Supplement: Supplementary file 2 — Additional file 2: Fig. S1. An illustration of the modules and operation procedures of Care Direct in client management. [file 12911_2022_2041_MOESM2_ESM.docx]

**Additional file 2: Fig S1. An illustration of the modules and operation procedures of Care Direct in client management**
