# Supplementary material for: Transition to a new nursing information system embedded with clinical decision support: a mixed-method study using the HOT-fit framework
Source: BMC Med Inform Decis Mak. 2022 Nov 28;22:310. doi: 10.1186/s12911-022-02041-y (PMC9703774; doi:10.1186/s12911-022-02041-y)
Supplement: Supplementary file 3 — Additional file 3. The topic guide (final version). [file 12911_2022_2041_MOESM3_ESM.docx]

**Additional file 3. The topic guide (final version)**

***Technical/task attributes***

1. What are the advantages and disadvantages of the new system compared with the original NIS?

2. What impact does the new system have on your clinical practice?

3. What improvements do you feel the new system have achieved?

***Organizational attributes***

1. technical support

*(For bedside nurses)*

What do you think of the support provided by technical staff?

*(For clinical/administrative leaders)*

What’s your experience with collaboration with technical staff?

2. Management support

*(For ward nurses)*

What do you think of the support and guidance from the management?

*(For nurses in administrative positions)*

What strategies do you use to motivate and supervise the nurses regarding system use?

What do you think of the support and guidance from the higher management?

3. What do you think of the training on system use?

***Human/individual attributes***

1. What do you feel about the clinical decision support provided by the system?

2. What are your expectations after the new system goes live?

***General***

1. What are the factors that affect your enthusiasm toward the new system?

2. What are your perceptions of the facilitators to using the system?
