# Supplementary material for: Transition to a new nursing information system embedded with clinical decision support: a mixed-method study using the HOT-fit framework
Source: BMC Med Inform Decis Mak. 2022 Nov 28;22:310. doi: 10.1186/s12911-022-02041-y (PMC9703774; doi:10.1186/s12911-022-02041-y)
Supplement: Supplementary file 4 — Additional file 4. Table S1. Responses to the NIS use behavior scale (n = 324). Table S2. Responses to the NIS effectiveness scale (n = 324). [file 12911_2022_2041_MOESM4_ESM.docx]

Additional file 4: Table S1. Responses to the NIS use behavior scale (n=324)

| Items |  | Options | | | | |
| --- | --- | --- | --- | --- | --- | --- |
|  | Average score | Strongly disagree | Disagree | Neutral | Agree | Strongly agree |
| I often use this system. | 3.91±0.88 | 4(1.23%) | 15(4.63%) | 72(22.22%) | 149(45.99%) | 84(25.93%) |
| I can use the system well. | 3.76±0.84 | 3(0.93%) | 14(4.32%) | 103(31.79%) | 143(44.14%) | 61(18.83%) |
| I'm used to the system. | 3.53±0.94 | 11(3.40%) | 24(7.41%) | 116(35.80%) | 127(39.20%) | 46(14.20%) |
| I actively learn the existing functions of the system. | 3.83±0.87 | 5(1.54%) | 17(5.25%) | 74(22.84%) | 161(49.69%) | 67(20.68%) |
| I actively learn the existing approach to using the system. | 3.90±0.84 | 6(1.85%) | 9(2.78%) | 71(21.91%) | 165(50.93%) | 73(22.53%) |
| I’d like to propose some new ways to use the system. | 3.62±0.87 | 7(2.16%) | 17(5.25%) | 114(35.19%) | 139(42.90%) | 47(14.51%) |
| I’d like to put forward new requests for the system. | 3.68±0.87 | 7(2.16%) | 15(4.63%) | 104(32.10%) | 148(45.68%) | 50(15.43%) |

Additional file 4: Table S2. Responses to the NIS effectiveness scale (n=324)

| Dimension | Items |  | Options | | | | |
| --- | --- | --- | --- | --- | --- | --- | --- |
|  |  | Average score | Strongly disagree | Disagree | Neutral | Agree | Strongly agree |
| System quality | Apart from the influence of Wi-Fi, the system response time to complete tasks is short with little lag. | 3.13±0.95 | 9(2.78%) | 75(23.15%) | 130(40.12%) | 84(25.93%) | 26(8.02%) |
|  | The information system can ensure the safety of patient information. | 3.79±0.88 | 1(0.31%) | 19(5.86%) | 104(32.10%) | 124(38.27%) | 76(23.46%) |
|  | The system can flexibly switch between various screens. | 3.05±0.95 | 17(5.25%) | 71(21.91%) | 131(40.43%) | 89(27.47%) | 16(4.94%) |
|  | The system is stable during operation | 2.96±0.99 | 18(5.56%) | 90(27.78%) | 123(37.96%) | 72(22.22%) | 21(6.48%) |
| Information quality | The information provided by the system can meet the types and contents of information required of nursing practice. | 3.33±0.98 | 10(3.09%) | 50(15.43%) | 124(38.27%) | 102(31.48%) | 38(11.73%) |
|  | The information provided by the system is consistent with real situation without errors. | 3.41±0.95 | 5(1.54%) | 51(15.74%) | 116(35.80%) | 110(33.95%) | 42(12.96%) |
|  | The system can obtain the required information within the timeframe required of nursing practice. | 3.27±0.99 | 14(4.32%) | 48(14.81%) | 137(42.28%) | 88(27.16%) | 37(11.42%) |
|  | The information provided by the system is continuous and dynamic, reflecting the change of patient condition. | 3.43±0.98 | 7(2.16%) | 41(12.65%) | 135(41.67%) | 88(27.16%) | 53(16.36%) |
|  | The decision support information such as diagnosis, intervention and outcome evaluation provided by the system is valuable and useful. | 3.26±0.98 | 14(4.32%) | 48(14.81%) | 137(42.28%) | 90(27.78%) | 35(10.80%) |
| Service quality | The hardware, software and other physical facilities of the system give users a good experience. | 3.02±1.03 | 24(7.41%) | 69(21.30%) | 136(41.98%) | 67(20.68%) | 28(8.64%) |

| Dimension | Items |  | Options | | | | |
| --- | --- | --- | --- | --- | --- | --- | --- |
|  |  | Average | Strongly disagree | Disagree | Neutral | Agree | Strongly agree |
|  | System development and maintenance personnel sincerely and timely solve the problems encountered during system use. | 3.52±0.98 | 10(3.09%) | 28(8.64%) | 124(38.27%) | 106(32.72%) | 56(17.28%) |
|  | The system use training can meet the needs of clinical practice. | 3.32±1.00 | 13(4.01%) | 47(14.51%) | 125(38.58%) | 100(30.86%) | 39(12.04%) |
|  | The system developers can understand the special needs of nurses. | 3.03±1.04 | 23(7.10%) | 74(22.84%) | 123(37.96%) | 77(23.77%) | 27(8.33%) |
| User satisfaction | I am satisfied with the overall performance of the system | 3.02±0.95 | 17(5.25%) | 74(22.84%) | 133(41.05%) | 84(25.93%) | 16(4.94%) |
|  | I am satisfied with the operability of the system. | 3.01±0.96 | 19(5.86%) | 73(22.53%) | 136(41.98%) | 79(24.38%) | 17(5.25%) |
|  | I am satisfied with the function of the system. | 3.06±0.96 | 17(5.25%) | 67(20.68%) | 143(44.14%) | 75(23.15%) | 22(6.79%) |
|  | I am satisfied with the decision support of the system. | 3.19±0.98 | 14(4.32%) | 58(17.90%) | 133(41.05%) | 89(27.47%) | 30(9.26%) |
|  | I am satisfied with the efficiency of the system. | 3.06±1.00 | 19(5.86%) | 70(21.60%) | 133(41.05%) | 77(23.77%) | 25(7.72%) |
| Net benefits | The system improves the support the process and outcomes of nursing practice. | 3.06±1.01 | 19(5.86%) | 72(22.22%) | 131(40.43%) | 75(23.15%) | 27(8.33%) |
|  | The system reduces the time required to complete the tasks. | 2.72±1.04 | 36(11.11%) | 110(33.95%) | 103(31.79%) | 59(18.21%) | 16(4.94%) |
|  | The system reduces the cost of nursing office supplies. | 3.29±1.04 | 14(4.32%) | 58(17.90%) | 114(35.19%) | 97(29.94%) | 41(12.65%) |
|  | The system has the ability of analysis and prediction in the nursing process. | 3.27±0.99 | 12(3.70%) | 56(17.28%) | 125(38.58%) | 95(29.32%) | 36(11.11%) |
|  | The system can streamline the delivery of nursing service. | 3.00±1.07 | 25(7.72%) | 80(24.69%) | 116(35.80%) | 75(23.15%) | 28(8.64%) |
