# Supplementary material for: Transition to a new nursing information system embedded with clinical decision support: a mixed-method study using the HOT-fit framework
Source: BMC Med Inform Decis Mak. 2022 Nov 28;22:310. doi: 10.1186/s12911-022-02041-y (PMC9703774; doi:10.1186/s12911-022-02041-y)
Supplement: Supplementary file 5 — Additional file 5. Table S3. Upgrade frequency of each module of the system from January 2021 to April 2022. [file 12911_2022_2041_MOESM5_ESM.docx]

Additional file 5: Table S3. Upgrade frequency of each module of the system from January 2021 to April 2022

| Module | | Update categories | | | | |
| --- | --- | --- | --- | --- | --- | --- |
|  |  | Debugging | | Optimization | | New contents added |
| Patient allocation | | 9 | | 1 | | 1 |
| Nursing assessment | | 9 | | 14 | | 5 |
| Nursing diagnosis | | 2 | | 1 | | 1 |
| Care planning | | 17 | | 9 | | 5 |
| Care implementation | | 21 | | 19 | | 12 |
| Specialized care | | 15 | | 35 | | 13 |
| Outcome evaluation | | 5 | | 5 | | 2 |
| Documentation editing | | 1 | | 2 | | 1 |
| Nursing shift handover | | 8 | | 10 | | 1 |
| Discharge care | | 1 | | 4 | | 0 |
| Nursing record query | | 6 | | 13 | | 5 |
| **Others** |  | |  | |  | |
| Main interface | | 7 | | 8 | | 0 |
| Process optimization | | 0 | | 3 | | 0 |
| Multi module issues | | 0 | | 3 | | 0 |
